# Complete Genome Sequence of an Enterovirus 71 Strain Isolated from the Cerebrospinal Fluid of a Child with Severe Hand-Foot-and-Mouth Disease in Yunnan, China, 2013

**DOI:** 10.1128/genomeA.00274-17

**Published:** 2017-05-11

**Authors:** Keqin Lin, Haihao Zhang, Wei Bai, Yiling Zhao, Hongbo Liu, Xiaoqin Huang, Zhaoqing Yang, Shaohui Ma

**Affiliations:** a Institute of Medical Biology, Chinese Academy of Medical Sciences, and Peking Union Medical College, Kunming, PR China; b Yunnan Key Laboratory of Vaccine Research Development on Severe Infectious Disease, Kunming, PR China

## Abstract

The complete genome sequence of the enterovirus 71 strain CSF15/YN/CHN/2013, first isolated from cerebrospinal fluid of a child in Yunnan, China, in 2013, was determined. According to the phylogenetic and homogeneity analyses, the isolate was assigned to subgenotype C4a.

## GENOME ANNOUNCEMENT

Human enterovirus 71 (EV71) belongs to the *Picornaviridae* family and is the main pathogen of hand-foot-mouth disease (HFMD) in young children and infants. EV71 infection generally results in a mild and self-limiting disease. But in some cases it can also result in aseptic meningitis, encephalitis, or even death (1). EV71 is a positive-sense, single-stranded RNA virus. The genome is approximately 7.4 kb, consisting of a 5′ untranslated region (UTR), structural polypeptide P1, nonstructural polypeptides P2 and P3, and a 3′ UTR. Beginning in 2008, EV71 has caused epidemic HFMD in China every year. All EV71 strains can be classified into 3 genotypes (A, B, or C) and 12 subgenotypes (A, B1 to B5, and C1 to C5) (2, 3). Understanding the molecular epidemiology of EV71 in regions where it is endemic is an important factor in the prevention and treatment of EV71-mediated HFMD.

To the best of our knowledge, the CSF15/YN/CHN/2013 strain was first isolated from a cerebrospinal fluid (CSF) sample, which was collected from a patient diagnosed with severe HFMD in Yunnan, China. The CSF sample was inoculated into rhabdomyosarcoma cell lines (RD cells) and propagated in up to three passages. EV71-infected cells were harvested and preserved at −80°C. After viral RNA was extracted, primer pairs 222 and 224 were used to amplify the partial VP1 gene (4) using a one-step reverse transcription (RT)-PCR method as described previously (5). The isolate was defined as EV71 using sequence comparison with sequences available in GenBank using the BLAST algorithm (http://www.ncbi.nlm.nih.gov/BLAST/). Then, seven synthetic oligonucleotide primer pairs, designed using previously published data, were used to amplify overlapping fragments that span the whole genome of EV71 by the RT-PCR method described previously (5). The whole genome sequence of strain CSF15/YN/CHN/2013 was established by assembling overlapping fragments using BLAST. Nucleic acid and protein sequence alignments were analyzed by Geneious Basic 5.6.5 software. The viral genome sequence of the strain CSF15/YN/CHN/2013 was composed of 7,404 nucleotides (nt), excluding the poly (A) tail. The 5′ UTR was found to be 741 nt, followed by an open reading frame (ORF) including the structural protein region P1 (2,586 nt), the functional protein regions P2 (1,734 nt) and P3 (2,259 nt), and the 3′ UTR (84 nt). The contents of A, U, G, and C were 27.1%, 24.7%, 23.9%, and 24.3%, with G+C content of 48.1%. A phylogenetic tree was constructed by use of Molecular Evolutionary Genetic Analysis (MEGA) version 6.06. The results of the phylogenetic analyses suggest that strain CSF15/YN/CHN/2013 belongs to subgenotype C4a.

### Accession number(s).

The complete genome sequence of CSF15/YN/CHN/2013 has been deposited in GenBank under the accession no. KY425527.

## References

[1] Huang PN, Shih SR 2014 Update on enterovirus 71 infection. Curr Opin Virol 5:98–104. doi:10.1016/j.coviro.2014.03.007. 24727707

[2] Chong P, Liu CC, Chow YH, Chou AH, Klein M 2015 Review of enterovirus 71 vaccines. Clin Infect Dis 60:797–803. doi:10.1093/cid/ciu852. 25352588

[3] Solomon T, Lewthwaite P, Perera D, Cardosa MJ, McMinn P, Ooi MH 2010 Virology, epidemiology, pathogenesis, and control of enterovirus 71. Lancet Infect Dis 10:778–790. doi:10.1016/S1473-3099(10)70194-8. 20961813

[4] Nix WA, Oberste MS, Pallansch MA 2006 Sensitive, seminested PCR amplification of VP1 sequences for direct identification of all enterovirus serotypes from original clinical specimens. J Clin Microbiol 44:2698–2704. doi:10.1128/JCM.00542-06. 16891480 PMC1594621

[5] Li H, Shao CW, Pan Y, Ke HX, Ma SH 2012 Complete genome sequence characteristics of human enterovirus 71 strain isolated in Yunnan, China [in Chinese]. Bing Du Xue Bao 28:108–113. 22519170

